# Cathepsin F is a potential marker for senescent human skin fibroblasts and keratinocytes associated with skin aging

**DOI:** 10.1007/s11357-022-00648-7

**Published:** 2022-09-03

**Authors:** Kento Takaya, Toru Asou, Kazuo Kishi

**Affiliations:** Department of Plastic and Reconstructive Surgery, Keio University School of Medicine, 35 Shinanomachi, Shinjuku-ku, Tokyo, 160-8582 Japan

**Keywords:** Cathepsin, Cellular senescence, Skin aging, Fibroblast, Keratinocytes, Senescence-associated secretory phenotype

## Abstract

Cellular senescence is characterized by cell cycle arrest and the senescence-associated secretory phenotype (SASP) and can be triggered by a variety of stimuli, including deoxyribonucleic acid (DNA) damage, oxidative stress, and telomere exhaustion. Cellular senescence is associated with skin aging, and identification of specific markers of senescent cells is essential for development of targeted therapies. Cathepsin F (CTSF) has been implicated in dermatitis and various cancers and participates in cell immortalization through its association with Bcl family proteins. It is a candidate therapeutic target to specifically label and eliminate human skin fibroblasts and keratinocytes immortalized by aging and achieve skin rejuvenation. In this study, we investigated whether CTSF is associated with senescence in human fibroblasts and keratinocytes. In senescence models, created using replicative aging, ionizing radiation exposure, and the anticancer drug doxorubicin, various senescence markers were observed, such as senescence-associated β-galactosidase (SA-β-gal) activity, increased SASP gene expression, and decreased uptake of the proliferation marker BrdU. Furthermore, CTSF expression was elevated at the gene and protein levels. In addition, CTSF-positive cells were abundant in aged human epidermis and in some parts of the dermis. In the population of senescent cells with arrested division, the number of CTSF-positive cells was significantly higher than that in the proliferating cell population. These results suggest that CTSF is a candidate for therapeutic modalities targeting aging fibroblasts and keratinocytes.

## Introduction 

Cellular senescence is a state of permanent cell cycle arrest related to telomere exhaustion, deoxyribonucleic acid (DNA) damage, chronic inflammation, mitochondrial dysfunction, or other causes [1]. Recent studies have shown that senescent cells secrete a series of inflammatory cytokines, chemokines, growth factors, and matrix remodeling factors that alter the local tissue environment and contribute to chronic inflammation and age-related diseases, such as cancer [2]. The accumulation of senescent cells has a detrimental effect on the organism via the senescence-associated secretory phenotype (SASP), providing a basis for the development of strategies to specifically eliminate senescent cells by a process known as “senolysis.” Since senescent cell elimination improves aging-related diseases and extends healthy life expectancy [3–5], this therapeutic strategy is attracting increasing attention.

First described by Hayflick et al. in 1961, cellular senescence is a phenomenon by which cell division in human fibroblasts is irreversibly arrested after approximately 50 passages [6]. Subsequently, senescent cells generated by irradiation with ionizing radiation [7] and the anticancer drug doxorubicin [8] have been analyzed. Increased senescence-associated β-galactosidase (SA-β-gal) activity; elevated protein expression levels of p16, p21, and p53; and decreased SIRT1 expression are markers of senescent cells [9]. Furthermore, levels of inflammatory cytokines such as IL-1A and IL-6 are elevated in SASP-expressing senescent cells compared with those in proliferating cells [10]. Senescent cells become immortalized despite the arrest of cell division by evading apoptosis via the Bcl family of apoptotic proteins [11].

SASP factors (cytokines, growth factors, and matrix metalloproteinases) secreted by immortalized senescent cells disrupt tissue metabolism locally and systemically, and this has been associated with disease-enhancing effects of senescent cells accumulating in senescent tissues. However, these phenotypes are generally used to identify cellular senescence, but do not label senescent cells. This is because the phenotypes are not restricted to senescent cells. Discovery of unique markers that function as senescence-associated autoantigens can facilitate senescent cell elimination and clearance via the immune system. One solution for eliminating these senescent cells is to focus on the fact that senescent cells differ significantly from proliferating cells in their pattern of protein expression, including cell surface proteins that can serve as markers and therapeutic targets. This strategy is similar to that used to selectively eliminate cancer cells [12]. So far, DPP4 [13] and PLAUR [14] have been identified as membrane proteins specifically expressed in senescent cells. Against this background, identification of a targetable senescence marker would complement interventions aimed at eliminating senescent cells.

The objective of this study was to investigate a candidate senescent cell-specific marker, cathepsin F (CTSF). Cathepsins are cysteine proteinases, which are major components of the lysosomal protein degradation system [15]. Recently, CTSF has been identified as a marker of cancer cells in cervical and gastric cancers [16, 17]. In addition, CTSF decreases Bcl-2 levels in the apoptosis pathway [18]. In contrast, in adipose stem cells, apoptosis is suppressed and cathepsin F expression is downregulated, thereby reducing radiation-induced dermatitis [19]. However, the association between CTSF expression and human fibroblasts in aging has not been thoroughly investigated. To fill this knowledge gap, we investigated the behavior of CTSF in a model of human keratinocytes and dermal fibroblast senescence. We hypothesized that CTSF is specifically expressed in association with the SASP in a senescent epidermal or dermal cell model. Furthermore, we confirmed the ability of CTSF to label senescent cells by expression profiles in vitro and in vivo.

Previous therapies targeting senescent cell surface antigens have been useful in improving the function of other organs [13, 14], but to date, this has not been achieved for skin rejuvenation. Our candidate, CTSF, may be a robust surface antigen on human skin fibroblasts or other constituent cells, making this study highly novel.

## Materials and methods

### Cell culture

Normal human dermal fibroblasts (NHDF; C-12300) and normal human epidermal keratinocytes (NHEK; C-12003) were obtained from PromoCell GmbH (Heidelberg, Germany). NHDF were grown and maintained in low-glucose Dulbecco’s Modified Eagle’s Medium (Wako Pure Chemical Industries, Osaka, Japan) supplemented with 10% fetal bovine serum (Thermo Fisher Scientific, Waltham, MA, USA) and 1% penicillin/streptomycin (Thermo Fisher Scientific). NHEK were cultured in a growth medium (PromoCell, C-20011) containing bovine pituitary extract, epidermal growth factor, insulin, hydrocortisone, epinephrine, transferrin, and CaCl_2_.

Replicative senescence (ReS) was defined as lack of cell growth for more than 2 weeks, i.e., cells with a cell population doubling level of 50–55. For ionized radiation-induced senescence, cells were exposed to 10 Gy of X-rays by the X-irradiator CellRad (Faxitron, Tucson, AZ, USA) and analyzed after 10 days. Control (proliferating) cells were mock irradiated by removal from the incubator, transport to the irradiator, and maintenance outside of the irradiator for the same period as the irradiated cells. For doxorubicin-induced senescence, cells were treated twice with 0.1 μM doxorubicin (Sigma-Aldrich, St. Louis, MO, USA) at 2-day intervals and analyzed after 7 days. SA-β-gal activity in cells was assessed using the Senescence β-Galactosidase Staining Kit from Cell Signaling (Danvers, MA, USA).

### Assessment of BrdU incorporation in fibroblasts by flow cytometry

Cells were incubated with BrdU at 37 °C for 24 h, collected, and incubated with the BrdU-FITC antibody (BrdUFlowEx FITC Kit; EXBIO Praha, a.s., Vestec, Czech Republic) for 30 min. FlowJo (Ver. 10.2) was used to analyze the data using a flow cytometer (BD Biosciences, Franklin Lakes, NJ, USA).

### Immunocytochemistry

Cells were placed on glass slides, fixed in acetone at room temperature (15–25 °C) for 5 min, and dried completely before staining. The cells were incubated overnight at 4 °C with an anti-cathepsin F antibody (PA5-87,925, Thermo Fisher Scientific) and anti-IL-6 antibody (ab6672, Abcam, Cambridge, UK) diluted 1:100 in phosphate-buffered saline (PBS). To confirm the specificity of the immunostaining, fetal bovine serum diluted to the same concentration was used instead of the primary antibody as a negative control. After washing three times with PBS, the slides were incubated with Alexa Fluor 488-conjugated goat anti-rabbit antibody and AlexaFluor555-conjugated donkey anti-goat antibody (Thermo Fisher Scientific) diluted 1:2000 in PBS for 1 h at room temperature. After incubation, nuclei were washed three times with PBS and counterstained for the visualization of nuclei using ProLong Gold Anti-fade Mountant containing 4′,6-diamidino-2-phenylindole (Thermo Fisher Scientific).

### Immunohistochemistry

Whole skin sample slides of 3- and 89-year-old human trunks were obtained from OriGene Technologies, Inc. (Rockville, MD, USA) under ethical considerations. Samples were collected after obtaining informed consent of anonymous donor patients. The procedures were compliant with American standards and applicable local ethical guidelines. Paraffin was dissolved in a slide heater (ThermoBrite; Leica Biosystems, Nussloch, Germany) at 65 °C for 30 min immediately before use. The slides were then deparaffinized by washing with xylene twice at room temperature (5 min per soak). Slides were soaked twice with 100% ethanol (3 min per soak) and then stepwise with 95%, 70%, and 50% ethanol (3 min each), and then rehydrated at room temperature. After antigen activation by heat, slides were incubated with 2% goat serum in PBS for 30 min at room temperature to block nonspecific binding sites. The slides were then incubated overnight at 4 °C with a 1:100 dilution of the same anti-CTSF antibody (in PBS) used for immunohistochemistry.

After washing three times with PBS, the slides were incubated with a 1:500 dilution of biotinylated rabbit anti-goat antibody (Vector Laboratories, Burlingame, CA, USA) in PBS for 1 h at room temperature.

The signal was amplified by the avidin-biotinylated peroxidase complex (ABC) method using the VECTASTAIN ABC Kit (Vector Laboratories) and 20 mg/dL 3,3′-diaminobenzidine solution (FUJIFILM Wako Pure Chemicals, Co., Osaka, Japan) for 1 to 3 min to develop color.

Sections were then washed once with running tap water for 5 min before nuclear counterstaining with Gill’s hematoxylin solution (Merck Millipore, Billerica, MA, USA) for 6 s at room temperature. Finally, sections were rinsed with tap water for 5 min, dehydrated with ethanol (twice with 95% and twice with 100%, 5 min each), rinsed with xylene three times, and sealed with Mount Quick Sealant (Takara Bio, Shiga, Japan).

Slides were observed using an integrated stereomicroscope (BZ-X800; KEYENCE, Osaka, Japan).

### RNA extraction and reverse transcription

Total RNA was extracted from cells using a monophasic solution of phenol and guanidine isothiocyanate (ISOGEN; Nippon Gene, Tokyo, Japan) according to the manufacturer’s instructions. Total RNA was mixed with random primers, reverse transcriptase, and a deoxynucleotide mixture (Takara Bio). The mixtures were incubated in a T100™ thermal cycler (Bio-Rad Laboratories, Inc., Hercules, CA, USA) at 25 °C for 5 min for annealing, 55 °C for 10 min for synthesis, and 80 °C for 10 min for thermal inactivation of reverse transcriptase to provide cDNA.

### Real-time quantitative polymerase chain reaction (RT-qPCR)

RT-qPCR was performed on an Applied Biosystems 7500 Fast Real-Time PCR System (Thermo Fisher Scientific). A total of 40 cycles were performed and the fluorescence of each sample was measured at the end of each cycle. The polymerase chain reaction (PCR) was performed in two major steps: (1) 95 °C for 3 s (denaturation) and (2) 60 °C for 30 s (annealing and extension). In the subsequent melting curve analysis, the temperature was increased from 60 to 95 °C and fluorescence was measured continuously. Gene expression was determined using primers for *CTSF* (assay ID: Hs00186901_m1), *Il-6* (Hs00985639_m1), *Il-1a* (Hs00174092_m1), *Il-8* (Hs00174103_m1), *p16*^*ink4a*^ (Hs00923894_m1), and *CEBPB* (Hs00270923_s1) (all from Thermo Fisher Scientific) and PCR master mix (Cat. No. 4352042; Applied Biosystems, Foster City, CA, USA) following the manufacturers’ instructions. *GAPDH* (Hs02786624_g1) was used as a control gene for normalization. The gene expression level in the proliferating cell population was used as the baseline, and fold change values were determined by the 2^−ΔΔCT^ method.

### Western blotting

Total protein was extracted from cells with lysate buffer (50 mM Tris–HCl (pH 8.0), 150 mM NaCl, 0.5% Nonidet P40, 0.5% sodium deoxycholate, and phenylmethylsulfonyl fluoride (all from FUJIFILM Wako Pure Chemical Co.)).

Each sample (40 μg) was electrophoresed on 10% polyacrylamide gels (Mini-PROTEAN TGX Precast Gels; Bio-Rad Laboratories, Inc.) and transferred to Trans-Blot Turbo Transfer System (Bio-Rad Laboratories, Inc.) onto polyvinylidene difluoride membranes (Millipore, Bedford, MA, USA).

After blocking with 3% nonfat milk for 2 h at room temperature, primary antibodies against CTSF (PA5-48,002; Thermo Fisher Scientific, 1:200), p21 (ab220206; Abcam, 1:100), CDKN2A/p16INK4a (EPR1473; Thermo Fisher Scientific, 1:200), SIRT1 (ab32441; Abcam, 1:200), and GAPDH (Santa Cruz Biotechnology, Santa Cruz, CA, USA, 1:2000) diluted with blocking solution were incubated overnight at 4 °C. The next day, the samples were incubated with the following secondary antibodies: donkey anti-goat IgG H&L (HRP) (ab6885; Abcam), goat anti-rabbit IgG H&L (HRP) (ab205718; Abcam), and goat anti-mouse IgG H&L (HRP) (ab205719; Abcam) at a 1:1000 dilution for 2 h at 37 °C. After washing, immunoreactive protein bands were visualized using an Electrochemiluminescence Detection Kit (Pierce Biotechnology, Rockford, IL, USA). Images of bands were obtained using a chemiluminescence imager (ImageQuant LAS4000mini; GE Healthcare, Chicago, IL, USA). The image analysis was performed using ImageJ (Ver. 1.53p, National Institutes of Health, Maryland, USA). Each experiment was repeated three times.

### Fluorescence-activated cell sorting (FACS)

Proliferating and senescent human skin fibroblasts were counted using a TC20 Cell Counter (Bio-Rad) and washed using FACS buffer (0.5% bovine serum albumin in PBS). After washing, human TruStain FcX (BioLegend, San Diego, CA, USA) was added to block the Fc receptor and cells were incubated with an anti-CTSF antibody (Thermo Fisher Scientific) for 10 min at 4 °C. Cells were then incubated with an Alexa Fluor 488-labeled rabbit anti-goat antibody (Thermo Fisher Scientific) for 15 min at 4 °C in the dark. Next, 7-amino-actinomycin D (7AAD) (Immunostep, S.L., Salamanca, Spain) was added and incubated at 4 °C for 15 min to label the photoreceptor cells. A FACS analysis was performed using FlowJo (version 10.2). Briefly, the background autofluorescence of the negative population was measured using an unstained control. 7AAD-viable cells were then gated, and the number of Alexafluor488-positive cells among them was counted.

### Statistical analysis

Statistical analyses were performed using GraphPad Prism (version 5.0; San Diego, CA, USA) or SPSS 22.0 (Chicago, IL, USA). Single end-point measures were compared using a non-paired sample *t*-test. Values of *P* < 0.05 were considered statistically significant.

## Results

### CTSF is specifically expressed in aging human dermal fibroblasts

**Fig. 1:**
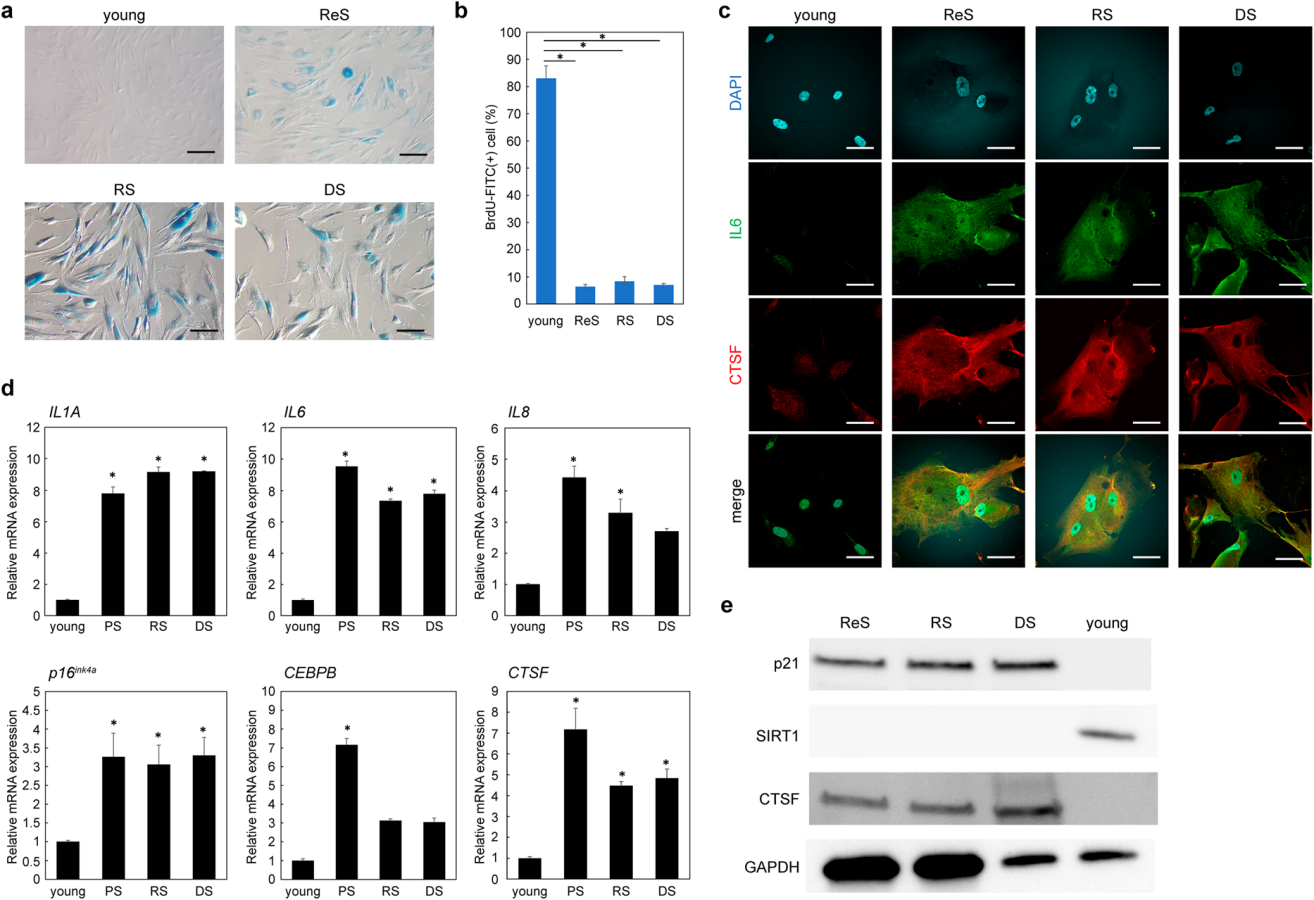
CTSF is upregulated in aged human skin fibroblasts. **a** SA-β-gal staining. Bar = 50 µm. **b** BrdU incorporation in proliferating and senescent cells. **c** Immunostaining of proliferating and senescent cells for CTSF and IL-6. Bar = 20 µm. **d** Gene expression of *CTSF* and SASP factors in proliferating and senescent cells. **e** Expression of CTSF protein in proliferating and senescent cells. *P < 0.05. CTSF, cathepsin F; DS, doxorubicin-induced senescence; ReS, replicative senescence; RS, radiation-induced senescence; SASP, senescence-associated secretory phenotype

To find new markers for senescent cells, we used three models of cellular senescence. In addition to the well-characterized proliferating human dermal fibroblasts (PDL 10) and cells that have undergone long-term culture (PDL 50), cells subjected to radiation-induced senescence and doxorubicin-induced senescence showed characteristic flattened and expanded morphologies and increased SA-β-gal activity (Fig. 1a). An analysis of BrdU uptake further showed that proliferative activity was significantly lower in senescent cells than in proliferating cells (young vs ReS, *P* = 0.000083; young vs radiation-induced senescence (RS), *P* = 0.000109; young vs doxorubicin-induced senescence (DS), *P* = 0.00008; Fig. 1b).

Immunostaining results also showed that CTSF was expressed in all three senescence models and these cells also expressed IL-6, an SASP factor (Fig. 1c). Furthermore, RT-PCR showed that the expression levels of *CTSF* as well as SASP factors, such as *IL1A*, *IL6*, *IL8*, *p16*^*ink4a*^ (*Cdkn2A*), and *CEBPB*, were upregulated in all senescent cell models compared to proliferating (young) cells (Fig. 1d). Western blotting also showed higher expression levels of CTSF in the senescent cell model than in control cells, along with increased expression of p21 in senescent cells and decreased expression of SIRT1 in proliferating cells (Fig. 1e).

### CTSF is specifically expressed in aging human epidermal keratinocytes

**Fig. 2:**
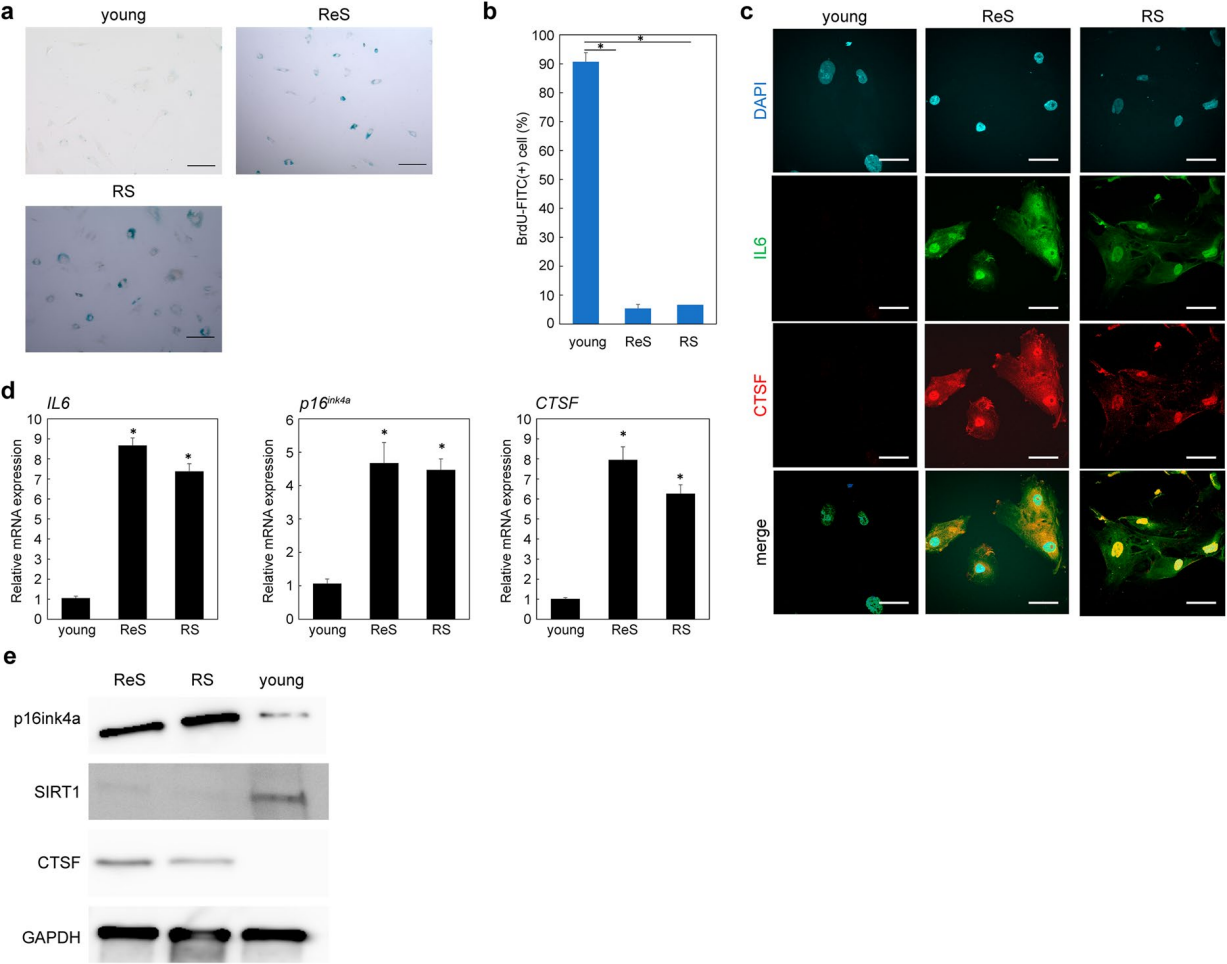
CTSF is upregulated in aged human skin keratinocytes. **a** SA-β-gal staining. Bar = 50 µm. **b** BrdU incorporation in proliferating and senescent cells. **c** Immunostaining of proliferating and senescent cells for CTSF and IL-6. Bar = 20 µm. **d** Gene expression of *CTSF* and SASP factors in proliferating and senescent cells. **e** Expression of CTSF protein in proliferating and senescent cells. *P < 0.05. CTSF, cathepsin F; DS, doxorubicin-induced senescence; ReS, replicative senescence; RS, radiation-induced senescence; SASP, senescence-associated secretory phenotype

We examined in vitro whether CTSF could be a new senescence marker in keratinocytes, which are representative of epithelial cells. Since only replicative senescence and radiation senescence have been reported in keratinocytes, we constructed a senescence model accordingly. We found an increase in the number of SA-β-gal-positive cells and a decrease in BrdU uptake in ReS and RS (young vs ReS, *P* = 0.00015; young vs RS, *P* = 0.00018; Fig. 2a,b). Immunostaining also showed CTSF positivity in senescent cells, consistent with IL-6 positivity (Fig. c), and RT-qPCR showed significantly elevated CTSF expression, consistent with increased expression of IL-6 and p16ink4a, representatives of SASP factors in keratinocytes (Fig. 2d). Even at the protein level, CTSF expression was elevated in aged keratinocytes (Fig. 2e). Thus, CTSF functions as a potential senescence marker not only in dermal fibroblasts but also in epidermal keratinocytes.

### CTSF is upregulated in the epidermis and dermis of aged human skin

**Fig. 3:**
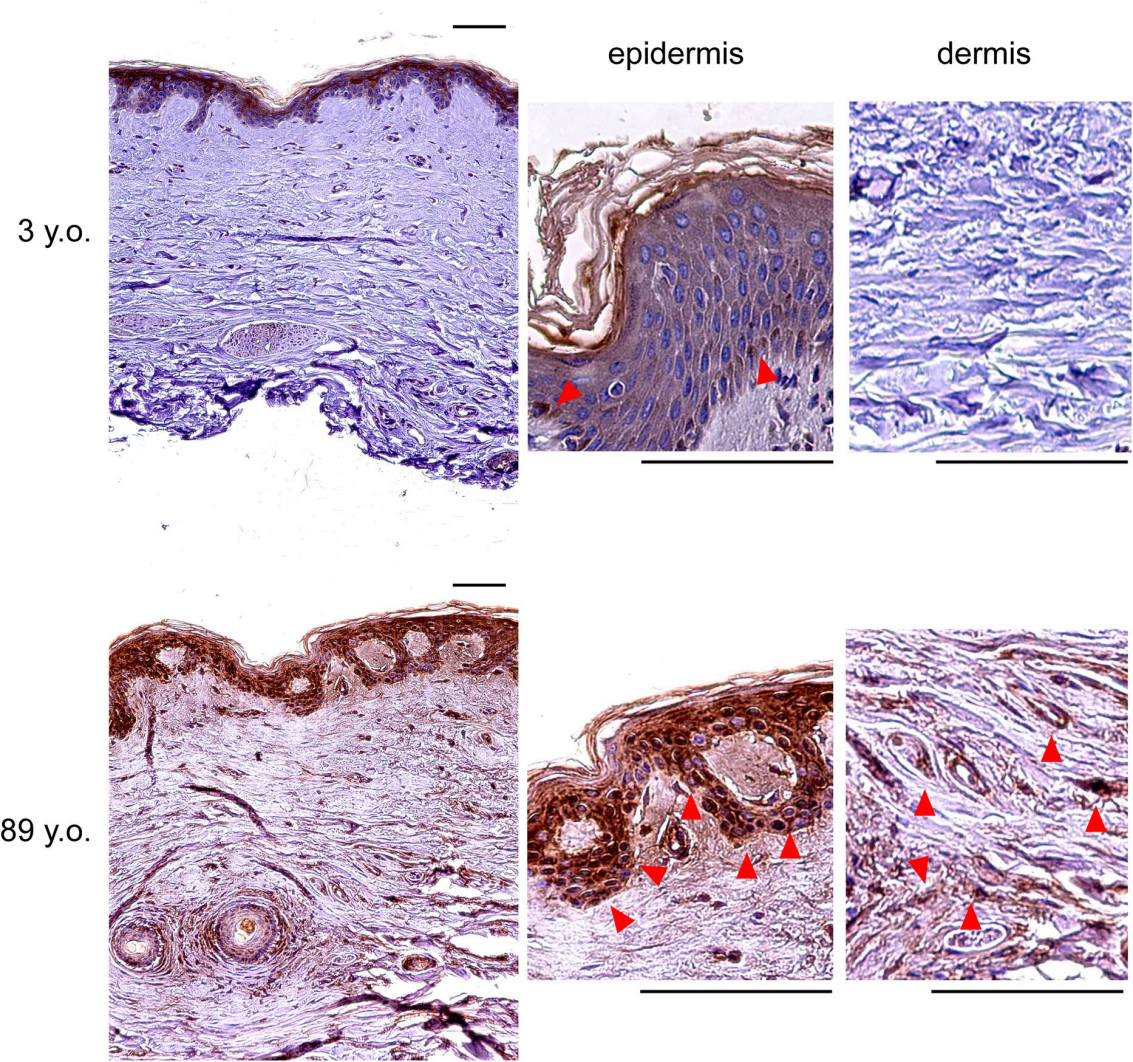
Expression of CTSF in human skin. In young skin tissue, only some cells in the epidermal layer are CTSF positive, but in old skin, many CTSF-positive cells are observed in all layers of the epidermis and dermis. Red arrows: CTSF-positive cells. Bar = 100 µm. CTSF, cathepsin F

Next, we observed CTSF functions in human skin as a marker of aging using immunostaining in human skin samples from patients aged 3 and 89 years. In aged human skin, all cells of the epidermis were CTSF positive. In contrast, in the epidermis of young human skin, only some CTSF-positive cells were found in the basal layer. Within the dermis, only few CTSF-positive cells were observed in young skin, whereas multiple such cells were observed in aged skin (Fig. 3). Thus, CTSF is suggested to be a marker of epidermal or dermal cells in aging skin in vivo.

### Selective targeting of senescent cells using CTSF antibodies

**Fig. 4:**
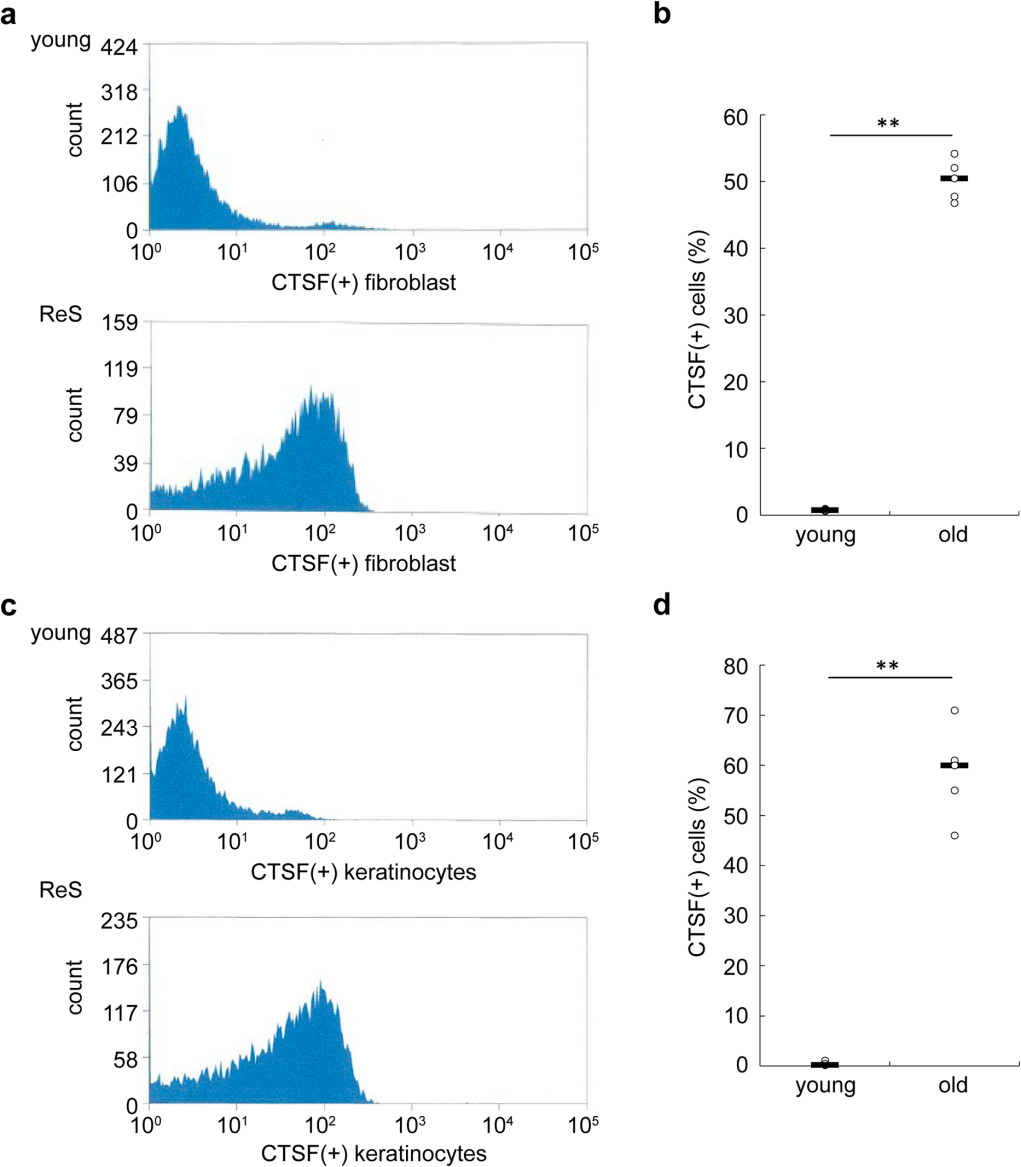
Sorting of senescent cells using an anti-CTSF antibody. **a** Sorting of CTSF-positive cells in proliferating and senescent human skin fibroblasts. **b** Comparison of the number of CTSF-positive fibroblasts. **c** Sorting of CTSF-positive cells in proliferating and senescent human skin keratinocytes. **d** Comparison of the number of CTSF-positive keratinocytes. Each data point represents a young (*n* = 3 total) or ReS (*n* = 3 total) subject; horizontal lines indicate the mean values. **P < 0.01. CTSF, cathepsin F; ReS, replicative senescence

Using FACS, we analyzed whether CTSF could label senescent cells in a cell population. In dermal fibroblasts, 50.28% cells were CTSF positive in the ReS (PDL50) population, whereas this was negligible in proliferating cells (PDL10), indicating a significant difference between the groups (*P* = 0.0000036) (Fig. 4a,b). Furthermore, CTSF-positive proliferating cells accounted for less than 1% of keratinocytes, whereas this value was 58.6% in the ReS population, with a significant difference between groups (*P* = 0.00014) (Fig. 4c,d). Thus, CTSF may be a candidate marker for labeling senescent cells in vitro.

## Discussion

In summary, we found that CTSF is potently upregulated in senescent human skin cells. In addition, senescent cells in the cell population were selectively targeted by anti-CTSF antibodies, providing a useful basis for the development of therapies to eliminate senescent cells in the skin.

SASP factors (cytokines, growth factors, and matrix metalloproteinases) elicit local and systemic inflammation by acting on tissue metabolism, and SASPs in aging tissues are associated with diseases [20,21]. Similarly, SASP-induced tissue senescence caused by the accumulation of senescent cells has been reported in the skin [22]. As the removal of senescent cells by genetic manipulation improves age-related disease states [3], therapies that selectively destroy senescent cells to achieve the same goal have been reported. The previously reported senescent cell eliminators ABT263 [11] and ABT737 [23] are BH3 mimetic inhibitors of anti-apoptotic proteins (Bcl-xL, Bcl-2, and Bcl-w) developed for cancer therapy. However, it is important to note that the inhibition of Bcl-xL has serious side effects. Other senescent cell-depleting agents, such as dasatinib and quercetin[24], induce apoptosis in a subset of senescent cells. In addition, strategies for targeting antigens expressed specifically in senescent cells include antibody-dependent cellular cytotoxicity using dipeptidyl peptidase-4 labeling [13] and T-cell therapy using urokinase-type plasminogen activator receptor [14]. In this context, CTSF as a targetable marker of senescence can complement interventions aimed at eliminating senescent cells.

CTSF, also known as CATSF/CLN13, is located at 11q133 [25]. The CTSF pro-region is very long and unique within the papain family of cysteine proteases and is predicted to share structural similarities with cysteine protease inhibitors of the cystatin superfamily [13]. The cathepsin family, to which CTSF belongs, contains 15 isoforms [26], some of which have long been implicated in aging. For example, cathepsin B is upregulated in aging fibroblasts in association with the cell cycle [27]. Alternatively, downregulation of endogenous cathepsin D results in accumulation of reactive oxygen species and accelerates aging [28]. Furthermore, it has been suggested that cathepsin D level is increased in spontaneous senescence and may be involved in oxidative stress and decreased proteasome activity [29–31].

Although the importance of elevated CTSF levels in senescent cells is unknown, a recent study has shown that the knockdown of CTSF expression markedly enhances cell proliferation in gastric cancer and simultaneously reduces the level of apoptosis, indicating that CTSF functions as a tumor suppressor in gastric cancer [17]. p53, another major suppressor, is upregulated in senescent cells [32], and CTSF may function similarly as a major suppressor in senescent cells in response to DNA damage.

Accumulation of senescent cells in the skin is largely responsible for the skin phenotype characteristic of aging. The appearance of typical signs of wrinkle formation and elastic fiber senescence correlates with increased levels of p16INK4a-positive fibroblasts in the dermis [33]. In addition, organ cultures obtained similar to aging fibroblasts have signs typical of skin aging over time, including impaired epidermal morphogenesis [34].

Rapamycin is an inhibitor of mTOR, a protein that regulates the cell cycle and is involved in skin fibroblast senescence through the regulation of SASP. Rapamycin inhibits the translation of the membrane-bound cytokine IL-1a and thereby, secretion of the inflammation-inducing SASP factor induced by interleukin [35]. Results of clinical studies conducted using topical application of rapamycin to the skin of elderly subjects showed reduction in the levels of p16INK4a-positive skin fibroblasts as well as the number of fine wrinkles and an increase in skin thickness and elasticity [36]. Furthermore, in a recent study, ABT-263 and ABT-737 induced selective clearance of aging dermal fibroblasts, regardless of the method of aging induction, and skin from aging mice treated with ABT-263 or ABT-737 showed increased collagen density, epidermal thickness, and keratinocyte proliferation and decreased senescence-associated secretory phenotype-associated factors such as MMP-1 and IL-6 [37]. Thus, removal of SASP-positive senescent cells from the skin by targeting CTSF could be applied for improving the properties of aging skin.

Importantly, it has recently been noted that the senescent cell population is heterogeneous and expresses different markers depending on the tissue and cell type [38]. In this context, consistent with cells expressing SASP factors, CTSF was upregulated in a cellular senescence model population of dermal fibroblasts and keratinocytes, two important cell types that comprise human skin. Since such a marker has not been reported previously, it may be an important antigen in the development of skin rejuvenation therapies targeting senescent cell removal.

A limitation of this study is that the mechanism by which the manipulation of CTSF gene expression affects the aging phenotype was not determined. siRNA-based knockdown of CTSF did not affect the SASP phenotype of aging human skin fibroblasts (data not shown); however, it did affect the SASP of aging cells (data not shown). Future studies should examine the impact of CTSF on adults using knockout mouse models. In addition, our results indicate that CTSF is effective with respect to human skin fibroblasts and keratinocytes and may contribute to the development of skin rejuvenation therapies, but no conclusions can be drawn about the aging of other types of cells or fibroblasts in other tissues. If CTSF is found to be similarly useful in fibroblasts of other tissues, it may contribute to the development of therapies to remove senescent cells in other organs. Since other models of senescent cells include oncogene-induced senescence [39], it will be necessary to examine whether CTSF expression is also applicable to other models of senescence/other cell tumors when considering its application to systemic organs.

In conclusion, we showed that CTSF is specifically expressed in senescent human dermal fibroblast cells and may be useful for the development of senolytic therapies.
